# Supplementary material for: Structure of F1-ATPase from the obligate anaerobe Fusobacterium nucleatum
Source: Open Biol. 2019 Jun 26;9(6):190066. doi: 10.1098/rsob.190066 (PMC6597759; doi:10.1098/rsob.190066)
Supplement: Supplemental Information [file rsob190066supp1.docx]

**Supplemental Information for:**

**Structure of the F_1_-ATPase from the obligatory anaerobic bacterium *Fusobacterium nucleatum***

**Jessica Petri^1,a^, Yoshio Nakatani^1,2,‡^, Martin G. Montgomery^3,‡^, Scott A. Ferguson^1^, David Aragão^4^, Andrew G.W. Leslie^5^, Adam Heikal^1,2,b^, John E. Walker^3^ and Gregory M. Cook^1,2^**

^1^ Department of Microbiology and Immunology, Otago School of Medical Sciences, University of Otago, Dunedin 9054, New Zealand_;_ ^2^ Maurice Wilkins Centre for Molecular Biodiscovery, The University of Auckland, Private Bag 92019, Auckland 1042, New Zealand ^3^ Medical Research Council Mitochondrial Biology Unit, Cambridge Biomedical Campus, Cambridge CB2 0XY, United Kingdom; ^4^Australian Synchrotron, 800 Blackburn Road, Clayton, Victoria 3168, Australia; ^5^ Medical Research Council Laboratory of Molecular Biology, Cambridge Biomedical Campus, Cambridge CB2 0QH, United Kingdom

**
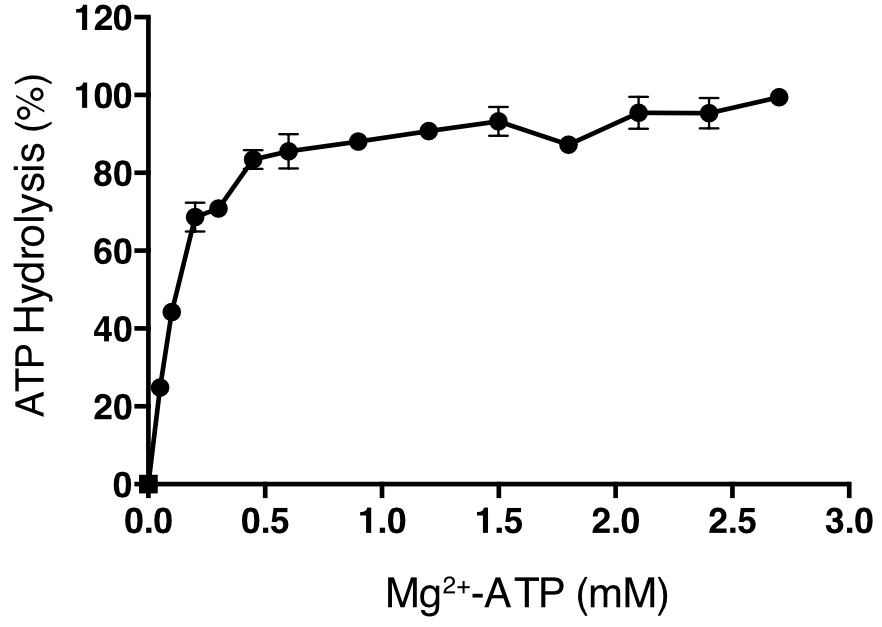
**

**Figure S1. Influence of Mg^2+^-ATP on ATP Hydrolysis by F_1_-ATPase from *F. nucleatum***

Effect of ATP at a constant Mg^2+^:ATP ratio of 2:1 in an ATP generating system. An activity of 100% corresponds to a specific activity of 4.59 U/mg; error bars represent the standard deviation of the mean from a biological triplicate. Where no error bars are shown, they are smaller than the diameter of the data points.


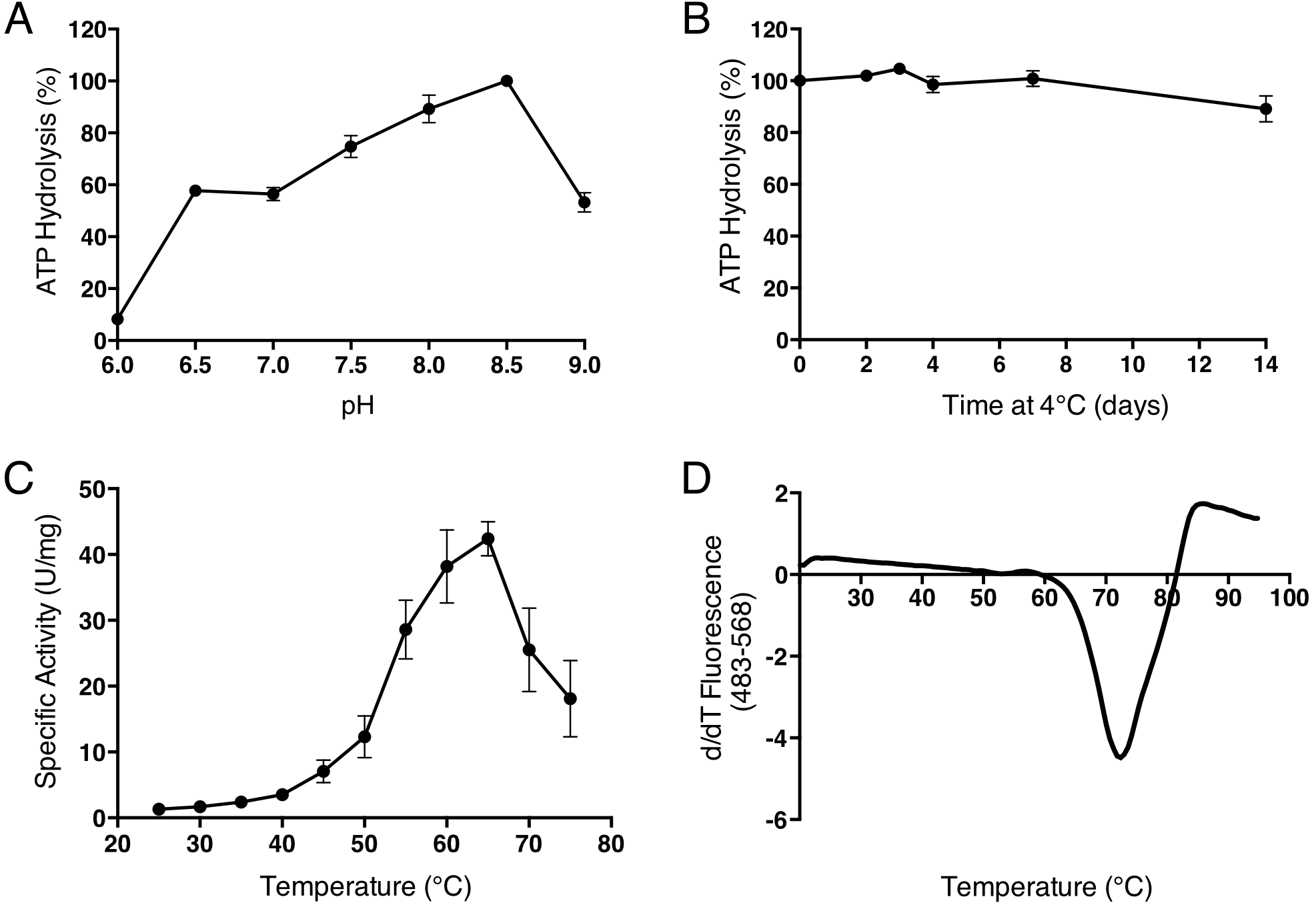


**Figure S2. Effects of pH and Temperature on the Activity and Stability of F_1_-ATPase from *F. nucleatum***

Part A, effect of pH as assayed in an ATP-regenerating system. An activity of 100% corresponds to a specific activity of 9.4 U/mg; part B, stability at 4˚C in buffer containing 20 mM Tris-HCl pH 8.0, 2 mM MgCl_2_, 100 mM NaCl and 10% [w/v] glycerol. ATP hydrolysis was monitored over 14-days. An activity of 100% corresponds to a specific activity of 7.1 U/mg; part C, effect of temperature on hydrolytic activity assayed by release of inorganic phosphate; part D, thermal denaturation of the enzyme. The trough corresponds to the midpoint of protein unfolding. One representative replicate is shown. Error bars represent the standard deviation of the mean from a biological triplicate. Where no error bars are shown, they are smaller than the diameter of the data points.

**
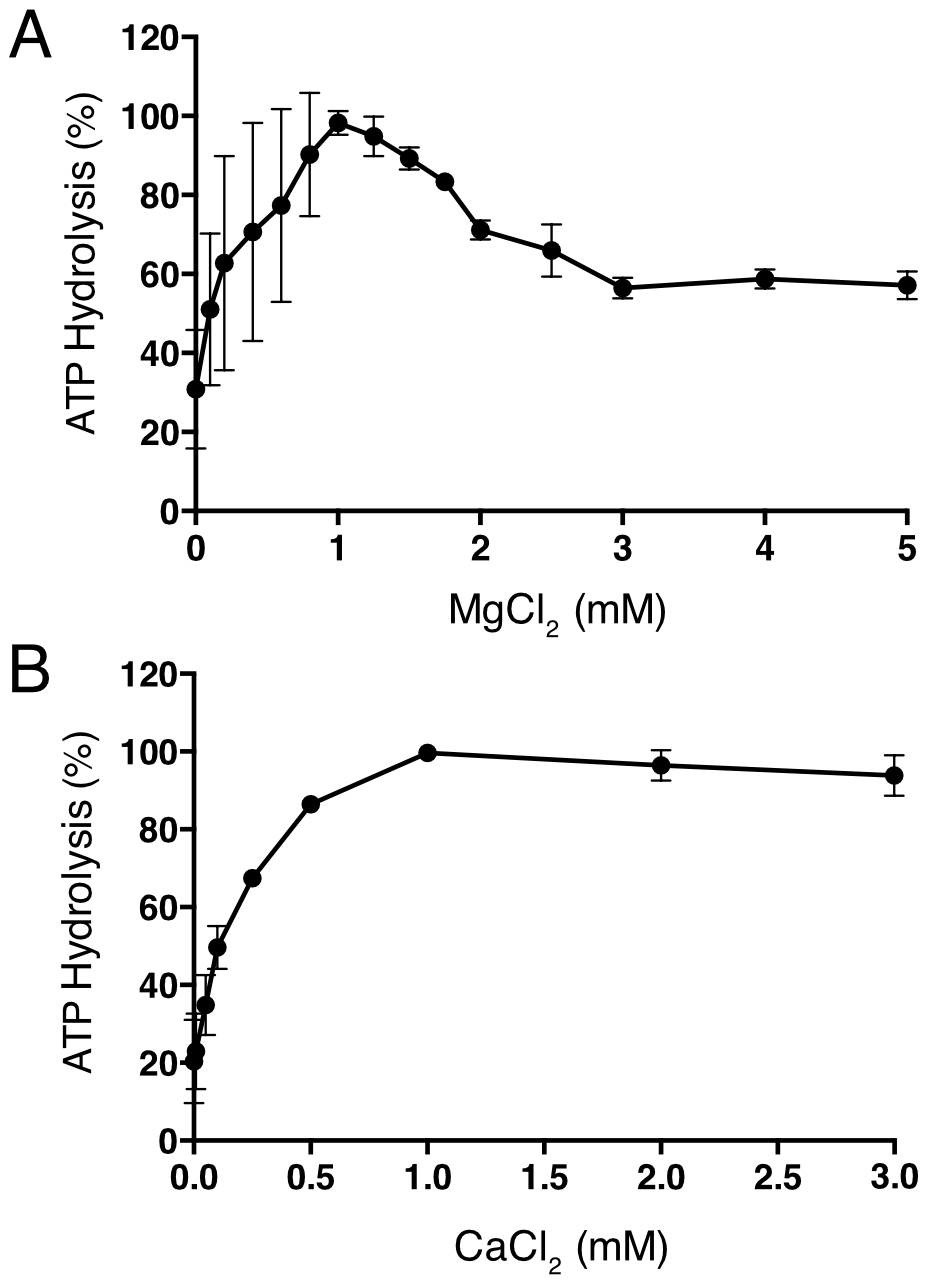
**

**Figure S3. Influence of MgCl_2_ and CaCl_2_ on ATP Hydrolysis by F_1_-ATPase from *F. nucleatum***

Parts A and B, effects on the hydrolytic activity of MgCl_2_ and CaCl_2_, respectively, determined by the release of inorganic phosphate. An activity of 100% corresponds to maximal specific activities of 4.4 and 7.8 U/mg for MgCl_2_ and CaCl_2_, respectively; error bars represent the standard deviation of the mean from a biological triplicate. Where no error bars are shown, they are smaller than the diameter of the data points.

**Table S1. Crystallographic Data Collection and Refinement Statistics**

| Parameter |  |
| --- | --- |
| Space group | P2_1_ |
| Unit cell dimensions a, b, c (Å) | 111.9, 200.2, 201.7 |
| Unit cell angles α, β, γ, (^o^) | 90, 102.2, 90 |
| Resolution range (Å) | 3.6-42.3 |
| High-resolution bin (Å) | 3.6-3.7 |
| Unique reflections | 97166 (4646) |
| Multiplicity | 6.1 (4.4) |
| Completeness (%) | 97 (94.6) |
| R_merge_^1^ | 0.21 (1.06) |
| <I/σ (I)> | 6.4 (1.5) |
| B factor, from Wilson plot (Å)^2^ | 86.3 |
| R factor^2^ (%) | 23.8 |
| Free R factor^3^ (%) | 28.0 |
| rmsd bonds (Å) | 0.003 |
| rmsd angles (^o^) | 0.7675 |

Parentheses denote the statistics for the high-resolution bin

^1^ R_merge_ = ∑*_h_*∑*_i_*|*I*(*h*)-*I*(*h*)*_i_*|/∑*_h_*∑*_i_I*(*h*)*_i_*, where *I*(*h*) is the mean weighted intensity after rejection of outliers.

^2^ R factor = ∑*_hkl_*∣∣*F_obs_*∣ - *k*∣*F_calc_*∣∣/∑*_hkl_*∣*F_obs_*∣, where *F_obs_* and *F_calc_* are the observed and calculated structure factor amplitudes, respectively.

^3^ R_free_ = ∑*_hkl⊂T_*∣∣*F_obs_*∣-*k*∣*F_calc_*∣∣/∑ *_hkl⊂T_*∣*F_obs_*∣, where *F_obs_* and *F_calc_* are the observed and the calculated structure factor amplitudes, respectively, and T is the test set of data omitted from refinement.


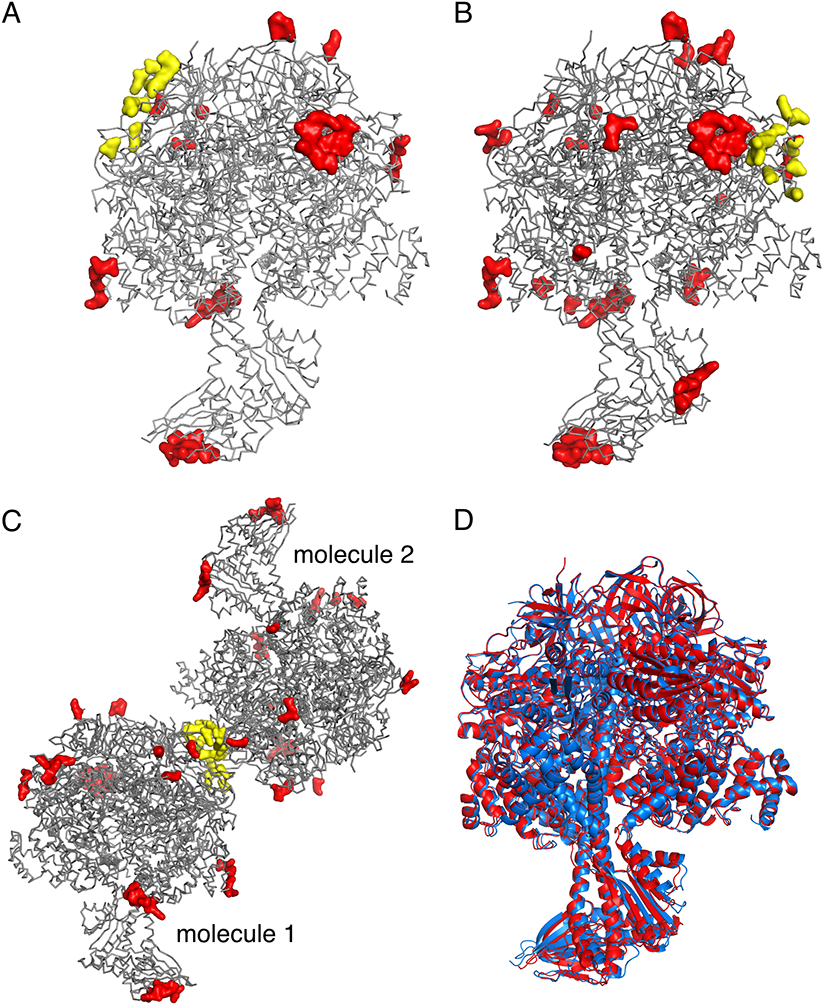


**Figure S4. Lattice Contacts in Crystals of F_1_-ATPase from *F. nucleatum***

Parts A and B, molecules 1 and 2, respectively, in grey ribbon with residues within 4 Å of either a symmetry mate or the second molecule in the asymmetric unit, in red or yellow surface representation, respectively; part C, arrangement of the molecules 1 and 2 in the asymmetric unit; part D, molecules 1 (blue) and 2 (red) superimposed via their crown domains. The foot of the central stalk is displaced by additional crystal contacts in molecule 2 around residues 108-113 of the γ-subunit.


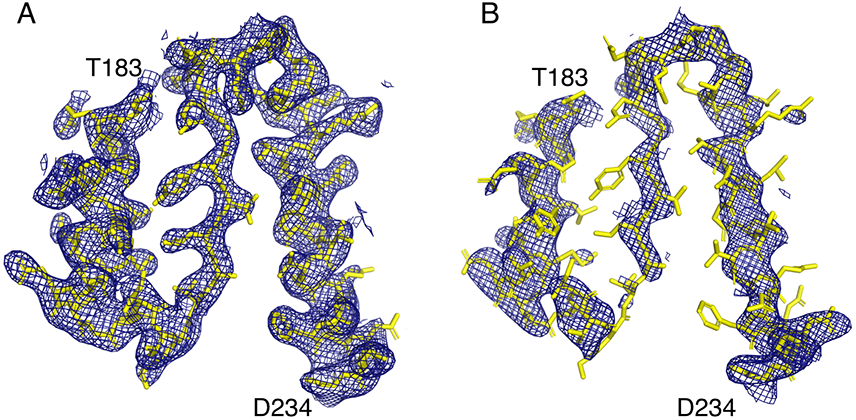


**Figure S5. Examples of Regions of the 2Fo-Fc Electron Density Map Derived by X-ray Analysis of Crystals of F_1_-ATPase from *F. nucleatum***

Electron density, contoured at 1.0 σ, representing residues 183-234 of the β_E_-subunit. Part A and B, molecules 1 and molecule 2, respectively, interpreted as an α-helix (residues 183-197), a β-sheet (residues 201-209) and an α-helix (214-234).

**Table S1. Comparison of the Structures of *F. nucleatum* Molecule 1 and Molecule 2**

| *F. nucleatum* | rmsd^a^ (Å) [residues matched] | | | |
| --- | --- | --- | --- | --- |
|  | F_1_ | α_3_β_3_ only | γ-subunit | ε-subunit |
| molecule 1 v molecule 2 | 1.4 [3196] | 0.5 [2783] | 0.3 [281] | 0.6 [132] |

**Table S3.** **Comparison of the Structure of the F_1_-ATPase from *F. nucleatum* with Those of Other F_1_-ATPases**

| Species | rmsd^a^ (Å) [residues matched] | | | |
| --- | --- | --- | --- | --- |
|  | F_1_ | α_3_β_3_ only | γ-subunit | ε-subunit |
| *M. smegmatis* (6foc) | 1.7 [3044] | 1.2 [2748] | 1.9 [191] | 5.0 [105] |
| *C. thermarum* (5ik2; molecule 1) | 2.4 [3191] | 1.2 [2785] | 2.8 [275] | 3.4 [130] |
| *S. oleracea* (6fkf) | 2.9 [3188] | 1.3 [2784] | 5.0 [272] | 2.3 [129] |
| *P. denitrificans* (5dn6) | 2.2 [3106] | 1.5 [2779] | 3.0 [254] | 1.4 [73] |
| Bovine (4asu) ^b^ | 2.3 [3025] | 1.6 [2766] | 3.8 [176] | 1.7 [80] |
| Bovine (2jdi) ^c^ | 2.8 [3035] | 1.6 [2765] | 3.7 [178] | 1.9 [88] |
| Bovine (4yxw) ^d^ | 3.3 [3102] | 1.6 [2764] | 3.3 [212] | 2.3 [126] |
| Bovine (1e79) ^e^ | 4.4 [3154] | 1.7 [2785] | 7.9 [240] | 2.5 [129] |
| *G. stearothermophilus* (4dx7) ^f^ | 5.3 [3045] | 3.0 [2687] | 3.5 [243] | 14.1 [118] |
| *E. coli* (3oaa; molecule 1) ^f^ | 6.6 [3170] | 2.9 [2768] | 3.0 [273] | 19.0 [128] |
| *G. stearothermophilus* (2e5y) ^g^ | - | - | - | 3.2 [127] |
| *E. coli* (1aqt) ^g^ | - | - | - | 2.7 [130] |
| *T. elongatus* (5zwl) ^h^ | - | - | - | 3.0 [129] |

^a^ calculated with α-carbons only; ^b^ catalytic dwell (crystallized in presence of phosphonate); ^c^ phosphate release dwell (high resolution ground state); ^d^ phosphate release dwell (crystallized in presence of thiophosphate); ^e^ covalently inhibited with dicyclohexyl-carbodiimide; ^f^ ε-subunit only in “up” position in intact F_1_-ATPase; ^g^ isolated ε-subunit in “down” position; ^h^ sub-complex of truncated γ-subunit and intact ε-subunit in the “down” position.


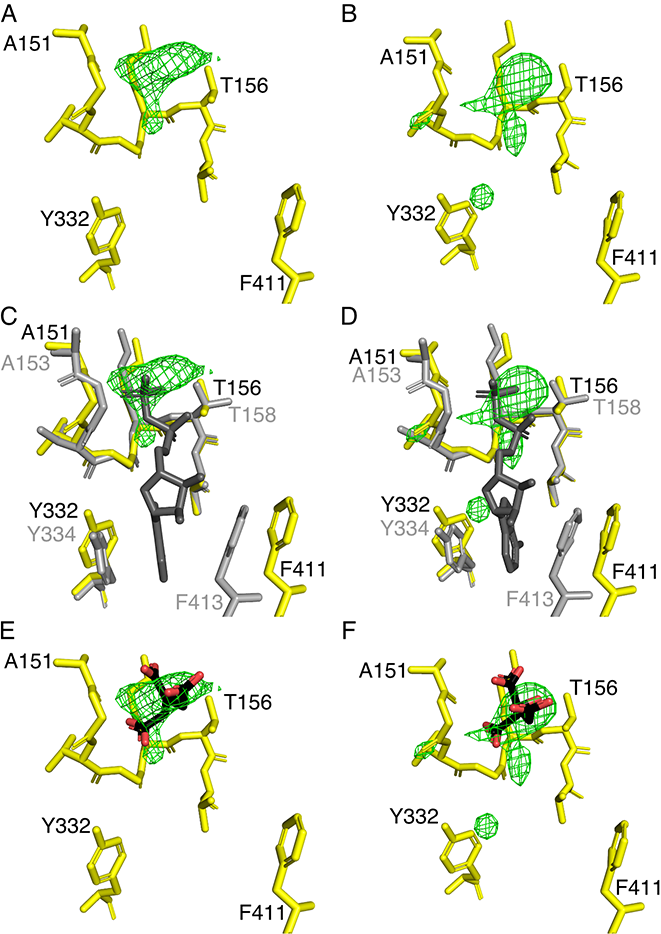


**Figure S6. Comparison of the Nucleotide Binding Sites in the β_E_-Subunits of the F_1_-ATPases from *F. nucleatum* and *C. thermarum***

In parts A and B, comparison of the difference density (green mesh, contoured to 3.0 σ) in the nucleotide binding site of the β_E_-subunits in molecules 1 and 2 in F_1_-ATPase from *F. nucleatum*, respectively; parts C and D, superimposition of the nucleotide binding site in the β_E_-subunits from *C. thermarum* (5ik2; grey) [51] with a bound ADP molecule and *F. nucleatum* (6q45; yellow); parts E and F, as in parts A and B but with a citrate molecule (black and red) modelled into the density.

**Table S4.** **Rotation Angles of γ-Subunits in F_1_-ATPases**

| Species | Angle (^o^) | Occupancy of β_E_-nucleotide binding site |
| --- | --- | --- |
| Bovine (1h8e) ^a^ | -14.8 | ADP, magnesium and sulfate |
| Bovine (2jdi and 4yxw) ^b^ | 0.0 | thiophosphate (4xyw only) ^b^ |
| *M. smegmatis* (6foc) ^c^ | 10.5 | low occupancy ADP possibly and phosphate (or sulfate) |
| *C. thermarum* (molecule 1; 5ik2) ^d^ | 11.7 | ADP and phosphate |
| *C. thermarum* (molecule 2; 5ik2) ^d^ | 13.2 | ADP and phosphate |
| *F. nucleatum* (molecule 1; 6q45) ^e^ | 19.7 | possibly citrate |
| *F. nucleatum* (molecule 2; 6q45) ^e^ | 19.1 | very low occupancy ADP possibly |
| *P. denitrificans* (5dn6) ^f^ | 27.0 | Unoccupied |
| Bovine (2v7q) ^g^ | 31.7 | Unoccupied |
| Bovine (4asu) ^h^ | 32.1 | ADP |
| *G. stearothermophilus* (4xd7) ^i^ | 38.4 | Sulfate adjacent to P-loop |
| *E. coli* (3oaa) ^i^ | 47.1-51.6 | Sulfate adjacent to P-loop |

^a^ crystallised in the presence of ADP and aluminium fluoride; ^b^ phosphate release dwell structures (high-resolution ground state structures of bovine F_1_-ATPase, and bovine F_1_-ATPase crystallized in presence of thiophosphate); ^c^ 2jdi and 4yxw were crystallized in the presence of a low concentration of ADP (40 μM). 4yxw was crystallised in the presence of 3 mM thiophosphate; ^d^ crystallised in the presence of 0.5 mM ADP; ^e^ crystallised in the presence of 0.5 mM ADP but with no nucleotide in the cryoprotection buffer; ^f^ crystallized in the presence of ATP only; ^g^ catalytic dwell (bovine F_1_-ATPase inhibited by IF_1_ 1-60; crystallized in presence of ATP only); ^h^ catalytic dwell (bovine F_1_-ATPase crystallized in presence of phosphonate and 1 mM ADP); ^i^ crystallized in absence of exogenous nucleotides.


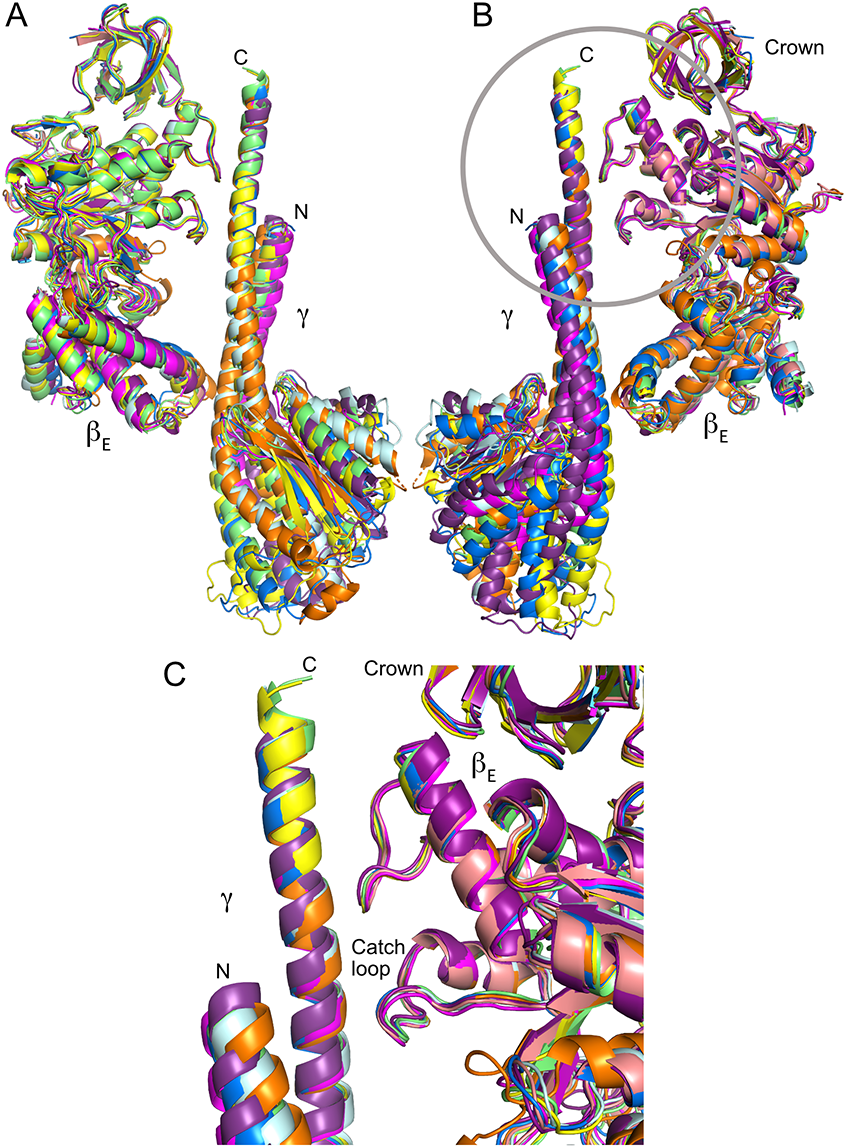


**Figure S7. Structures of β_E_- and γ-Subunits in F_1_-ATPases from Several Species Aligned via Their Crown Domains.**

The structures are as follows: *F. nucleatum* (6q45; blue), *E. coli* (3oaa; purple), *P. denitrificans* (5dn6; pink), *C. thermarum* (5ik2; yellow), *M. smegmatis* (6foc; green), bovine phosphonate (4asu; magenta), AlF_4_ (1h8e; orange), thiophosphate (4yxw; cyan). Parts A and B, side views of γ-subunits and associated β_E_-subunits. In part B, the view is rotated by 180^o^ relative to part A. Part C, magnified version of the encircled region in part B. In the superimposed structures, the final four turns of the C-terminal α-helix of the γ-subunit, and the crown (residues 1-95; *F. nucleatum* numbering) and “catch loop” regions (residues Y298-D306; *F. nucleatum* numbering) of the β-subunit are in very similar positions. In contrast, the C-terminal domains of the β-subunits and the regions of the γ-subunits below the “catch” adopt a range of different positions. This suggests that these interactions are retained across both the phosphate release and catalytic dwells and help to apply tension in the coiled coil domain of the γ-subunit during rotary catalysis. Mutations to the “catch loop” affect the catalytic activity of the enzyme [64]. It is possible that the interactions dissociate at some point around the ATP binding dwell.


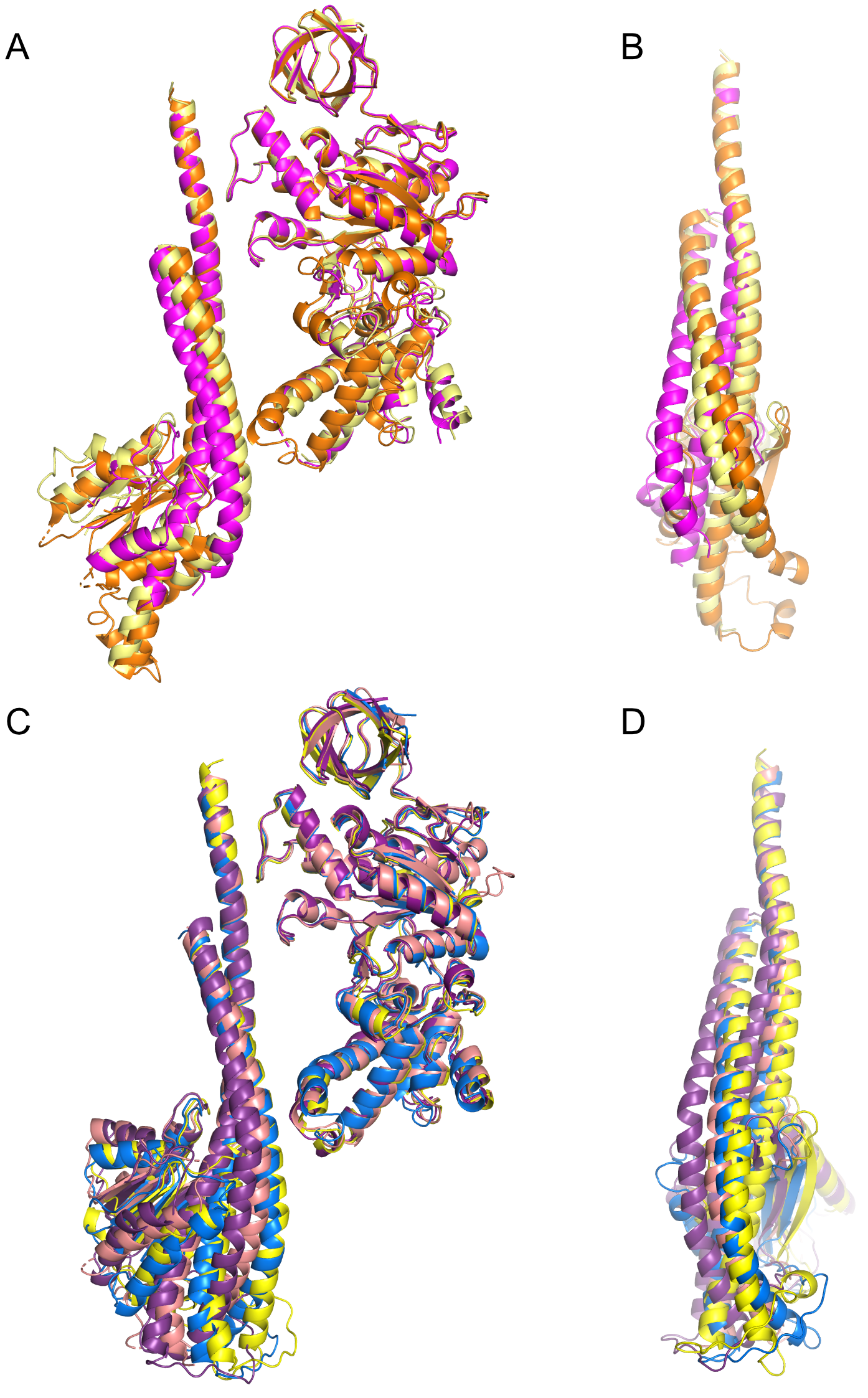


**Figure S8. Rotational Positions of the γ-Subunit in Structures of Bovine and Bacterial F_1_-ATPases.**

Parts A and B, bovine structures; parts C and D, bacterial structures. In A and B, the structure of bovine F_1_-ATPase crystallised in the presence of phosphonate (4asu) (magenta) represents the catalytic dwell, and the structure (light yellow) of bovine F_1_-ATPase inhibited by the phosphate analogue, thiophosphate (4yxw), represents the phosphate release dwell. The γ-subunit in the structure of bovine F_1_-ATPase inhibited by ADP-AlF_4_ (1h8e) is shown in orange. It is not clear which catalytic intermediate is represented by this structure, but it is not the catalytic dwell as its rotational position lies slightly ahead of that in the phosphate release dwell. Parts C and D, the structures of the ATP synthase from *P. denitrificans* (5dn6; pink) and of F_1_-ATPase from *E. coli* (3oaa; purple) representing the catalytic dwell. The γ-subunit in the structure of the F_1_-ATPase from *C. thermarum* (5ik2; yellow) is close to the position of the γ-subunit in the structure representing the mitochondrial phosphate release dwell. The γ-subunit in the structure of the F_1_-ATPase from *F. nucleatum* (6q45; blue) lies between this position and that of the γ-subunit in the catalytic dwell.


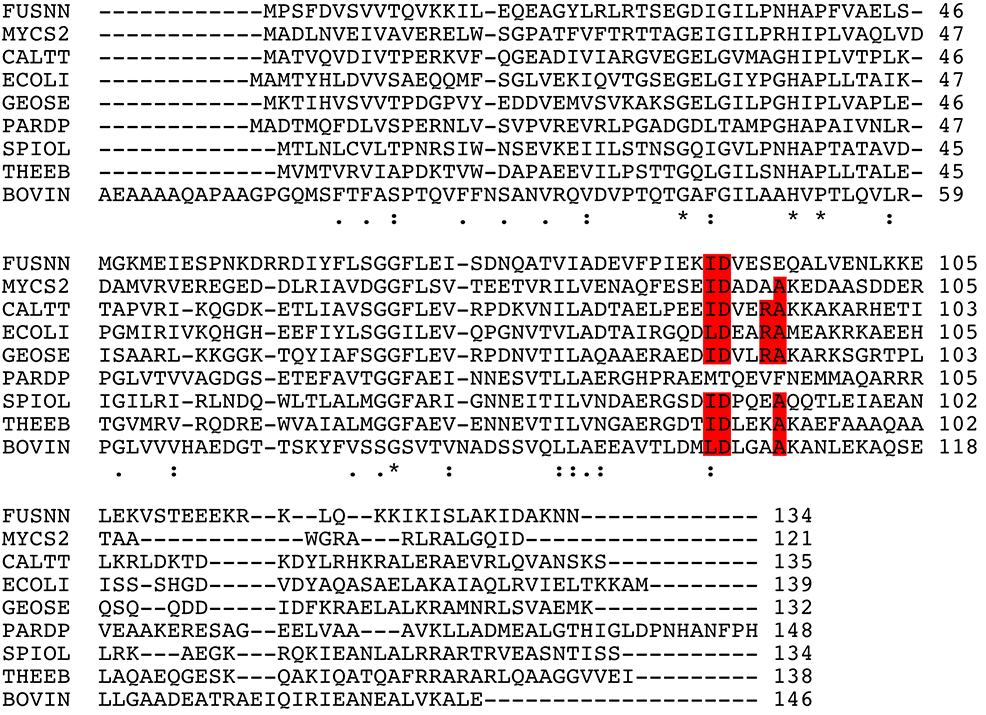
 **Figure S9. Alignment of Sequences of the ε-Subunits of ATP Synthases from Various Species**

FUSNN, *F. nucleatum*; MYCS2, *M. smegmatis*; CALTT, *C thermarum*; ECOLI, *E. coli;* GEOSE, *G. stearothermophilus*; PARDP, *P. denitrificans*; SPIOL, *Spinacia oleracea*; THEEB, *Thermosynechococcus elongatus*; BOVIN, bovine mitochondria. The residues marked in red are required for binding an ATP molecule to the ε-subunits in *C. thermarum, E. coli*, *and G. stearothermophilus*.
